# Familial cluster of COVID-19 infection from an asymptomatic

**DOI:** 10.1186/s13054-020-2817-7

**Published:** 2020-03-27

**Authors:** Jinjun Zhang, Sijia Tian, Jing Lou, Yuguo Chen

**Affiliations:** 1 School of Medicine, Shandong University, Jinan, 250012 Shandong China; 2 Beijing Emergency Medical Center, Beijing, 100031 China; 3 Emergency Department, Qilu Hospital, Shandong University, Jinan, 250012 Shandong China; 4 Shandong Provincial Clinical Research Center for Emergency and Critical Care Medicine, Jinan, 250012 Shandong China

Since December 2019, the first case of a novel coronavirus (COVID-19) infection pneumonia was detected in Wuhan, and the outbreak has been spreading rapidly in the world. As of February 18, 2020, a total of 73,332 cases of confirmed COVID-19 infection have been detected in the world as reported by the WHO [1, 2]. Given that the asymptomatic persons are potential sources of COVID-19 infection [3], we report a familial cluster case of five patients infected with COVID-19 from an asymptomatic confirmed case in Beijing. We obtained the data of patients, which included demographic, epidemiological, and clinical features; chest radiography; laboratory test; and outcomes. Laboratory confirmation of COVID-19 was detected in the first hospital admission and verified by the Beijing Center for Disease Control and Prevention (CDC). An asymptomatic case was defined as a laboratory-confirmed COVID-19 infection case who was afebrile and well. We enrolled the family that had five patients in total with COVID-19 infection who were transferred by the Beijing Emergency Medical Service (EMS) from January 24 to 27, 2020, to the designated hospitals for special treatment. Clinical outcomes were followed up to February 29, 2020.

The familial cluster of five patients (index patient to patient 4) was infected with COVID-19, and just the index patient had been to Wuhan, who had no symptoms before his family members started to get sick one after another. On January 19, the index patient, a 48-year-old male, came back to Beijing from Wuhan. He invited his nephew (patient 3) for dinner. On January 22, patient 3, a 32-year-old man, became ill with continuous high fever and fatigue; the highest body temperature was 40.2 °C; he first visited a hospital on January 24. Two nasopharyngeal swabs sample were obtained and were found to be positive for COVID-19 on the real-time reverse transcription-polymerase chain reaction (RT-PCR) assay. Before he went to the hospital, his uncle (index patient) was informed that his relative in Wuhan had been infected with COVID-19. On January 23, the index patient’s wife, patient 1, a 45-year-old female, had a fever of 37.8 °C too. For the reason that his relative in Wuhan and nephew had been detected to be infected with COVID-19, his family visited a hospital together and tested the RT-PCR for COVID-19, the results of which were all positive (Fig. 1). The chest radiograph of patient 2 was normal, and it is noteworthy that she was an asymptomatic too. The index patient’s and his wife’s (patient 1) chest radiograph demonstrated ground glass opacities (Fig. 2). Then, the family were transferred by EMS to the designated hospitals for special treatment and isolated on January 27. On the same day, the nephew’s mother (patient 4) got a fever of 38.4 with joint pain; after being confirmed with COVID-19 infection, she was transferred to the designated hospital too. On February 18, 2020, index patient to patient 2 were discharged, the index patient had no fever along with dry cough, and patient 2 had no clinical symptoms during hospitalization, while patients 3 and 4 were still in hospital.

**Fig. 1:**
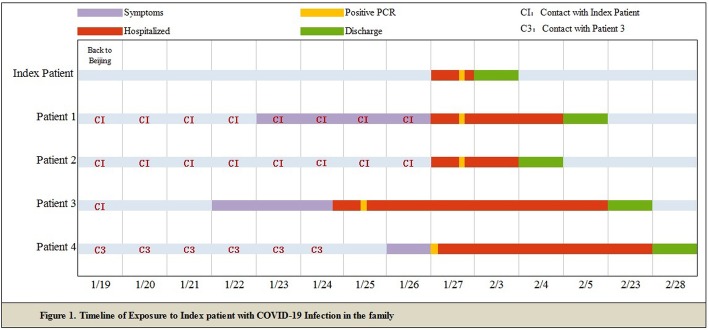
Timeline of exposure to index patient with COVID-19 infection in the familial cluster case

**Fig. 2:**
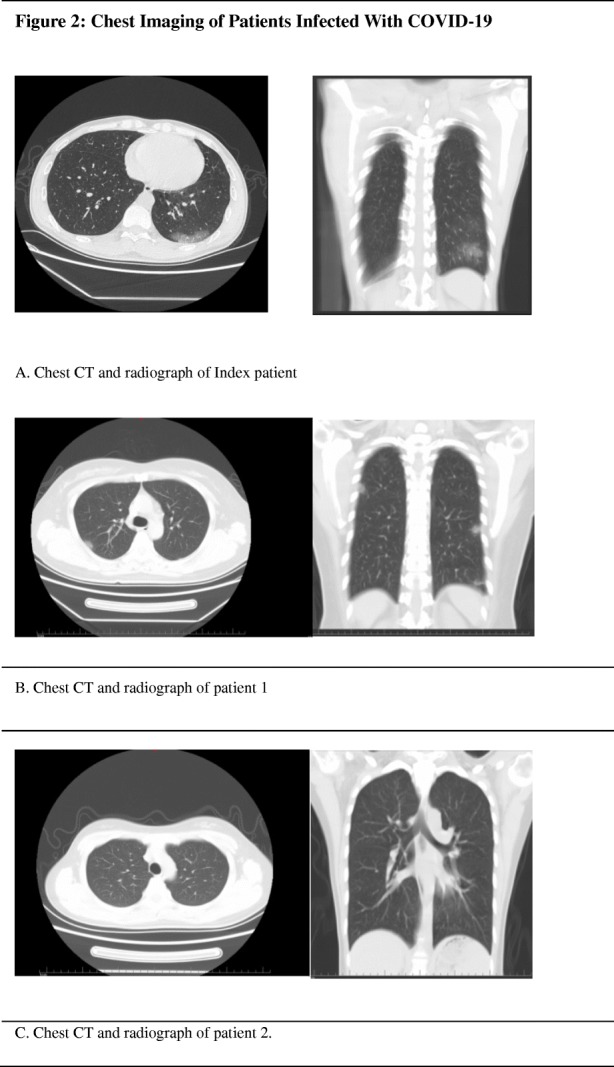
Chest imaging of patients infected with COVID-19

Finally, the familial cluster infected with COVID-19 has been reported in homes or hospital, especially without obvious symptoms [4]. According to our recent study, 5% of COVID-19 infection were asymptomatic cases [5]. If the asymptomatic cases cannot be found or isolated appropriately for medical observation, they will spread the virus to other close contacts quickly. Therefore, to identify and control the asymptomatic cases, as well as early quarantine for their close contacts, especially in families are important measures to prevent transmission of the COVID-19 infection.

## Data Availability

Not applicable.
